# Quantitative analysis of replication-related mutation and selection pressures in bacterial chromosomes and plasmids using generalised GC skew index

**DOI:** 10.1186/1471-2164-10-640

**Published:** 2009-12-30

**Authors:** Kazuharu Arakawa, Haruo Suzuki, Masaru Tomita

**Affiliations:** 1 Institute for Advanced Biosciences, Keio University, Fujisawa, 252-8520, Japan; 2 Department of Population Medicine and Diagnostic Sciences, College of Veterinary Medicine, Cornell University, Ithaca, NY 14853, USA

## Abstract

**Background:**

Due to their bi-directional replication machinery starting from a single finite origin, bacterial genomes show characteristic nucleotide compositional bias between the two replichores, which can be visualised through GC skew or (C-G)/(C+G). Although this polarisation is used for computational prediction of replication origins in many bacterial genomes, the degree of GC skew visibility varies widely among different species, necessitating a quantitative measurement of GC skew strength in order to provide confidence measures for GC skew-based predictions of replication origins.

**Results:**

Here we discuss a quantitative index for the measurement of GC skew strength, named the generalised GC skew index (gGCSI), which is applicable to genomes of any length, including bacterial chromosomes and plasmids. We demonstrate that gGCSI is independent of the window size and can thus be used to compare genomes with different sizes, such as bacterial chromosomes and plasmids. It can suggest the existence of different replication mechanisms in archaea and of rolling-circle replication in plasmids. Correlation of gGCSI values between plasmids and their corresponding host chromosomes suggests that within the same strain, these replicons have reproduced using the same replication machinery and thus exhibit similar strengths of replication strand skew.

**Conclusions:**

gGCSI can be applied to genomes of any length and thus allows comparative study of replication-related mutation and selection pressures in genomes of different lengths such as bacterial chromosomes and plasmids. Using gGCSI, we showed that replication-related mutation or selection pressure is similar for replicons with similar machinery.

## Background

DNA replication makes up a significant proportion of the bacterial cell cycle, especially in fast-growing bacteria where chromosomes undergo multiple rounds of replication in order to compensate for a short generation time [1]. Therefore, bacterial chromosomes are structured by the requirement to be an efficient medium for replication [2]. Eubacterial species typically have circular chromosomes that are partitioned into two replichores by one finite set of a symmetrically located replication origin and terminus [3]. Accordingly, many genomic features exhibit characteristic replication-related organisation, including the nucleotide compositional bias, distribution of signal oligonucleotides such as Chi sites [4,5] and KOPS motifs [6,7], as well as gene positioning and strand preference [8]. Nucleotide compositional asymmetry in the leading and lagging strands has been extensively studied using GC skew analysis, which calculates the excess of C over G normalised to the GC content ([C-G]/[C+G]) along the chromosome [9,10]. In many bacterial genomes, GC skew graphs "shift" their polarity between the two replichores, and thus the shift points of GC skew correspond to the replication origin and terminus. Analysis of the GC skew of a bacterial chromosome is therefore useful for the prediction of its replication origin and terminus [11] and, subsequently, its leading and lagging strands. The putative position of the replication origin predicted by computational methods based on GC skew is frequently used to define the first base position of circular genome sequences in many genome projects as an accurate and effective alternative to experimental means. Moreover, the polarisation of nucleotide composition is suggested to affect the replication-directed architecture of genomes. This includes the aforementioned replication-oriented sequence elements and gene orientation [13]; therefore, the degree of strand-specific mutational bias observed with GC skew analysis can be used as a reference for mutation or selection pressures that a genome receives due to the replication machinery [12-15].

Bacterial species exhibit highly diverse GC skew [16]. Many fast-growing bacteria show extremely biased GC skew, whereas only weak skew can be discerned in the chromosomes of slow-growing bacteria [17-19]. Therefore, the prediction of the replication origin with GC skew could be erroneous in genomes with only weak skew, requiring a quantitative confidence measure of GC skew strength. In order to allow comparative study of the degree of GC skew in bacterial genomes, we have previously reported the GC skew index (GCSI), which quantifies the strength of GC skew in given bacterial chromosomes and can be used as a confidence measure for GC skew-based predictions or for the comparative study of replication-related mutation or selection pressures in bacterial chromosomes [20]. The GCSI ranges from 0 to 1 and is calculated as an arithmetic mean of two indices: spectral ratio (*SR*) and *dist*. *SR *is the signal/noise (S/N) ratio of the 1 Hz signal in the Fourier power spectrum of a GC skew graph; it captures the fitness of the shape of the GC skew graph to be partitioned into two segments of opposite polarity having equal length (a discrete sine curve) [21], and *dist *measures the Euclidean distance between the two vertices in cumulative GC skew graphs. *SR *is essential for accurate quantification of a weak GC skew whose *dist *is affected by local regions of biased nucleotide content, such as large insertions. In order to eliminate the effects of biased nucleotide composition in coding regions, the GCSI is calculated with a fixed number of windows (4096, considering an average gene length of 1 kbp and a genome size of 2 to 4 Mbp). This use of a fixed number of windows limits the applicability of the GCSI to bacterial chromosomes and does not allow it to be used for shorter sequences, such as plasmids. Many plasmids are circular DNA molecules that exhibit nucleotide compositional asymmetry. GC skew is therefore frequently utilised for the prediction of replication origins in plasmids, which creates a need for extended applicability of the GCSI.

Circular plasmids can be categorised into two groups according to their replication machineries: theta and rolling circle replication (RCR). Theta replication requires the Rep protein and characteristic origins as well as DNA polymerase I from the host bacterium [22]. When there is only one origin of replication, theta replication results in two replichores of opposite polarity due to bi-directional replication forks, and hence these plasmids exhibit GC skew. Therefore, the shift points of the GC skew are indicative of the positions of the replication origin and terminus. The other type of replication, RCR, requires the RepABC family of proteins, and replication occurs through strand displacement [23-25]. In RCR, one of the two strands is always the template, and therefore plasmids that undergo RCR usually do not show significant GC skew. Instead, RCR plasmids show continuously biased nucleotide composition, resulting in linear cumulative GC skew, as opposed to the V-shaped graph observed in genomes with GC skew that indicates the existence of clear shift points.

It has been suggested that any genetic elements that reproduce inside the cell (chromosomes, plasmids, and phages) using the same replication machinery might have the same nucleotide composition and that recently acquired elements with unusual nucleotide compositions would drift towards the average nucleotide composition of the host genome by amelioration [26,27]. To investigate the evolution of plasmids in their hosts, comparisons have been made at the levels of GC content [28,29] and dinucleotide composition [30,31], but not from the viewpoint of replication strand asymmetry.

To this end, here we report a novel quantitative measure of GC skew strength called the generalised GC skew index (gGCSI) that is independent of window size and is therefore applicable to comparative studies of genomes of any length. Using this new index, we show discriminant criteria for the replication machinery of plasmids and the correlation of the degree of replication-related mutation or selection pressures in the host chromosome and plasmids.

## Results and Discussion

### Principle and Design of gGCSI

The original GCSI required the use of 4096 windows for optimal computation in bacterial genomes, but this fixed number of windows made GCSI only applicable to genomes larger than approximately 400 kbp; thus, each window contained at least 100 bp. The use of sliding windows is a simple means for increasing the number of windows, but this is technically just the moving average, which therefore diminishes the degree of GC skew and is not a solution to the problem. The limitations of the original GCSI were derived from the dependence of *SR *and *dist *on the number of windows; therefore, in order to generalise the GCSI to be applicable for smaller genomic elements, such as plasmids, we have made three modifications.

First, *SR *and *dist *were replaced with the normalised measure *SA *(spectral amplitude) and the normalised distance of the maximum and minimum vertices in the cumulative GC skew graph, *dist*(*norm*). Window-size dependence of *SR *was primarily due to the variation in basal noise levels depending on the number of windows, so the gGCSI is calculated simply using the amplitude of the 1-Hz Fourier power spectrum, without taking the S/N ratio. Because the distribution of spectral amplitude is non-linear, unlike *SR*, the exponentially regressed and thus linearised value for the 1-Hz spectrum is defined as *SA*. The other measure, *dist*, proportionally changes according to the number of windows, so it is linearly normalised as *dist*(*norm*).

Second, the gGCSI is defined as the geometric mean of *SA *and *dist*(*norm*), instead of the arithmetic mean utilised in the original GCSI. The arithmetic mean results in a relatively large value when only one of the two indices exhibits a large value; the use of a geometric mean instead ensures a balance between them.

Third, the statistical significance of the calculated gGCSI can be tested using the *z*-score and the *p*-value. Although the gGCSI is independent of the number of windows, the use of very few windows produces more uncertain results compared with when a sufficient number of windows are used for the calculation. In order to provide confidence measures in such cases, the *p *value of the gGCSI is obtained by repeatedly calculating the gGCSI using randomly shuffled input GC skew data series. Because the randomised iterations were statistically confirmed to be normal, a *z*-score and a corresponding *p*-value are given to the gGCSI to indicate its significance.

### Performance validation of the gGCSI

In order to test the applicability of the gGCSI to genomes of different sizes, we investigated the effects of the number of windows on the resulting values of the GCSI. First, we checked the effects in detail using the complete genome sequence of *Escherichia coli *K12 MG1655 (NC_000913), as shown in Table 1. The old GCSI, as well as the values of its contributing variables, *SR *and *dist*, increase proportionally with the number of windows, whereas the new gGCSI, *SA*, and *dist*(*norm*) show only small changes (standard deviation of 0.003 for gGCSI) as the number of windows changes, especially when more than 32 windows are used. Window independence of the gGCSI was further tested using bacterial chromosomes and plasmids of different sizes. Randomly sampled genomes, including the *Bacillus subtilis *chromosome (4.2 Mbp), *Mycoplasma genitalium *chromosome (0.58 Mbp), *Borrelia burgdorferi *cp32 plasmid (31 Kbp), *Staphylococcus aureus *pT181 plasmid (4.4 Kbp), and *Lactobacillus plantarum *pWCFS102 plasmid (2.3 Kbp), are shown in Table 2. In all of these genomes, the gGCSI showed only negligible changes when different numbers of windows from 8 to 32768 were used. The standard deviation of gGCSI values calculated with these windows was consistently low in all 1448 genomes used in this work: the 99.5% quantile was 0.035, and the lower 95% mean was 0.005, whereas for the GCSI, the values were 1.205 and 0.180, respectively. These results indicate that the gGCSI is independent of the window size and can be used to compare genomes with different sizes, such as bacterial chromosomes and plasmids.

**Table 1:** Comparison of GCSI and gGCSI values with different numbers of windows in the *Escherichia coli *K12 genome

number of windows	GCSI	*SR*	*dist*	gGCSI	*SA*	*dist(norm)*
8	0.0059	69.65	0.11	0.0884	472.84	58.77
16	0.0069	80.82	0.24	0.0902	478.82	60.33
32	0.0063	70.77	0.52	0.0963	483.45	67.16
64	0.0091	99.03	1.05	0.0965	484.12	67.35
128	0.0139	145.27	2.13	0.0971	483.97	68.11
256	0.0183	176.85	4.27	0.0973	483.97	68.40
512	0.0258	223.43	8.61	0.0978	485.00	68.90
1024	0.0368	269.47	17.16	0.0976	485.07	68.64
2048	0.0573	342.96	34.49	0.0980	486.53	68.97
4096	0.0953	453.39	69.04	0.0981	487.22	69.04
8192	0.1648	594.02	138.34	0.0984	488.97	69.17
16384	0.2932	744.70	277.35	0.0987	490.72	69.34
32768	0.5380	881.89	557.37	0.0989	490.30	69.67

**mean ± SD**	0.0978 ± 0.156	319 ± 271	85 ± 162	0.0964 ± 0.003	485 ± 5	67 ± 3

**Table 2:** gGCSI values of genomes with different sizes calculated with varying numbers of windows

	chromosomes		plasmids		
name	*B.subtilis*	*M.genitalium*	cp32	pT181	pWCFS102
**acession**	**NC_000964**	**NC_000908**	**NC_000952**	**J01764**	**NC_006376**

**size (bp)**	**4,214,630**	**580,076**	**30,800**	**4,439**	**2,365**

					

**8**	0.2093	0.1391	0.4758	0.2650	0.3949

**16**	0.2096	0.1388	0.4552	0.2674	0.4153

**32**	0.2139	0.1361	0.4399	0.2590	0.4501

**64**	0.2137	0.1341	0.4377	0.2762	0.4218

**128**	0.2142	0.1319	0.4349	0.3213	0.4305

**256**	0.2142	0.1290	0.4304	0.3432	n.a.

**512**	0.2143	0.1280	0.4267	n.a.	n.a.

**1024**	0.2141	0.1286	0.4299	n.a.	n.a.

**2048**	0.2141	0.1282	0.4327	n.a.	n.a.

**4096**	0.2141	0.1276	n.a.	n.a.	n.a.

**8192**	0.2144	0.1276	n.a.	n.a.	n.a.

**16384**	0.2147	0.1260	n.a.	n.a.	n.a.

**32768**	0.2145	0.1266	n.a.	n.a.	n.a.

					

**mean ± SD**	0.2135 ± 0.002	0.1309 ± 0.005	0.4403 ± 0.016	0.2887 ± 0.035	0.4225 ± 0.020

Although the gGCSI is independent of the window size, in practice a sufficiently large window size should be chosen such that it is not affected by the local nucleotide compositional bias. In most genomes, a window size of 1000 bp, which corresponds to the average length of coding genes, is sufficient. This leads to the use of 512 to 4096 windows in bacteria for optimal performance, considering the distribution of genome size in the range of 0.5 to 5 Mbp. However, for small plasmids that are only several kilobases in size, the use of 1000 bp windows results in only 4 or 8 windows, which is not sufficient for the calculation of *SA*. Because there is a trade-off between window number and size, the use of 16 to 32 windows of more than 100 bp is desirable for these small genomes.

In order to identify the optimal window size, we further calculated gGCSI using number of windows from 8 to 32768 in all bacterial genomes used in this work, and identified the windows size where the change in gGCSI value is minimum compared to adjacent window counts. For example, in Table 1, window number of 4096 has the least difference with the next window counts (0.0001 difference with 2048 windows and 0.0003 difference with 8192 windows). As shown in Supplemental Figure S1 [see Additional File 1], the median of optimal window number in all bacteria is 1024, which corresponds to the median of 2511 bp/window. Therefore, if a genome is sufficiently large, use of 1024 windows (2511 bp/window) produces the most accurate gGCSI value.

**Figure 1:**
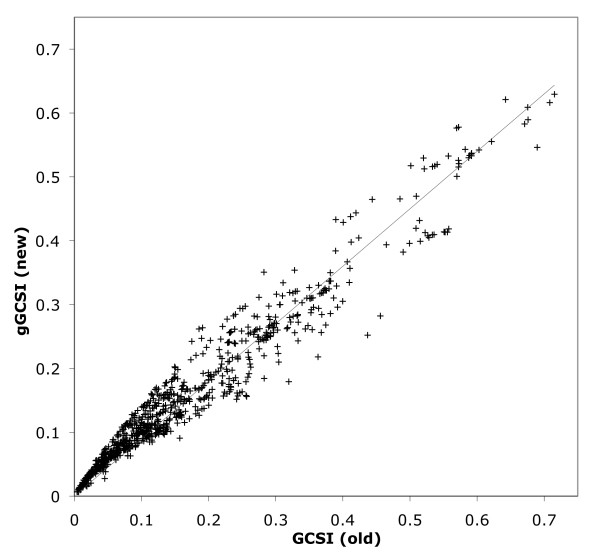
**Correlation of old and new GC skew index (GCSI) values**. Plot of GCSI (X-axis) and gGCSI (Y-axis) values for 822 bacterial chromosomes with 4096 windows.

Although the basic concept of integrating the Fourier power spectrum to capture the "shape" of the GC skew graph and the Euclidean distance between base compositions of leading and lagging strands remain unchanged in the gGCSI, this new index introduces several new calculation methodologies compared with the original GCSI, such as the use of a geometric mean and the calculation of *SA *without taking the S/N ratio. To test whether this new index can be used interchangeably with the original index, we have plotted the gGCSI value against the GCSI value for 822 complete bacterial chromosomes, using 4096 windows for the calculation of both indices (Figure 1). The two indices are highly correlated (Pearson product moment correlation coefficient, r = 0.993; Spearman rho rank correlation coefficient, *ρ *= 0.997), and therefore several criteria identified in a previous analysis (e.g., visible GC skew when GCSI > 0.1 and the absence of GC skew when GCSI < 0.05) can be applied to the gGCSI.

*SA *and *dist *are generally correlated, and majority of the genomes exhibit *dist/SA *ratio of around 0.184 (Supplemental Figure S2 [see Additional File 1]. However, this ratio varies by about 10-fold among the genomes, so that the geometric mean better captures the balance between the two indices than the arithmetic mean: (10*x *+ *x*)/2 = 5.5*x*, whereas . When GC skew continuously exists along one strand of the genome and does not shift its polarity, the strand results in extremely high *dist *while *SA *is low, deviating from the above *dist*/*SA *ratio. The genomes of *Pseudoalteromonas haloplanktis *TAC125 and *Halorhodospira halophila *SL1 are good examples for such continuously biased genomes, that show gGCSI < 0.1 with geometric mean, but exceed this threshold when calculated with arithmetic mean. This deviation is more pronounced with RCR plasmids that have the same non-shifting GC skew. Sixteen RCR plasmids used in this work showed gGCSI > 1.0 (with maximum of 1.544) when calculated with arithmetic mean, but the use of geometric mean limits to only one genome exceeding gGCSI > 1.0, with 1.069.

### Difference in GC skew strength between eubacteria and archaea with different types of replication machinery

**Figure 2:**
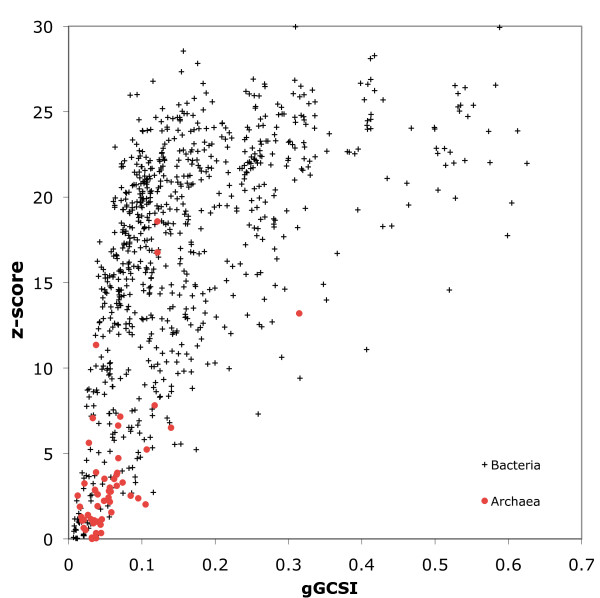
**Difference in GC skew strength between eubacteria and archaea**. Plot of gGCSI (X-axis) and z-score (Y-axis) for chromosomes of 710 eubacteria (black crosses) and 53 archaea (red closed circles) with 512 windows. Most of the archaeal chromosomes are located in the lower left corner, where gGCSI < 0.1 and z-score < 5.

As an application of the comparative capabilities of gGCSI, we investigated the effects of replication machinery on the degree of genomic compositional asymmetry. Genomic polarity in circular eubacterial genomes is attributed to bi-directional replication machinery starting from a finite single origin of replication, and thus GC skew is not observable in most archaeal genomes that contain multiple replication origins [32]. We have plotted the gGCSI values and corresponding z-scores for 822 eubacteria and archaea using 512 windows (Figure 2). Archaeal chromosomes represented by closed red circles are clustered around the lower left corner where gGCSI < 0.1 and z-score < 5, indicating the lack of selection pressure caused by bi-directional replication. Of the top ten archaeal chromosomes with high gGCSI values, only seven were significant (p < 0.01), including two human intestinal archaea *Methanobrevibacter smithii*(gGCSI = 0.315, z = 13.5) and *Methanosphaera stadtmanae *(gGCSI = 0.117, z = 7.06) that are reported to have visible GC skew, suggesting a single origin of replication for each [33]. Two *Halobacterium *species (gGCSI = 0.121, z = 18.2 and 17.1) had significantly high gGCSI values; for these species, multiple replication origins were suggested by computational analyses [34,35], but experimental validation through insertion of putative origins into non-replicating plasmid confirmed only one to be active *in vivo *[36]. *Pyrococcus horikoshii *(gGCSI = 0.140, z = 7.01) and *Pyrococcus abyssi *(gGCSI = 0.074, z = 3.11), for which the existence of only a single origin of replication has been extensively studied [37-41], also had significantly high gGCSI values. Although *Methanococcus aeolicus *(gGCSI = 0.107, z = 4.62) has no published evidence suggesting or confirming a single origin of replication, its gGCSI score suggests a high likelihood of bi-directional replication, which is supported by the V-shaped cumulative GC skew graph (Supplemental Figure S3 [see Additional File 1]). These results indicate that the gGCSI score, together with the statistical significance indicated by the z-score, can successfully distinguish differences in replication machinery between archaea and bacteria. The overall difference in the distributions of eubacteria and archaea could be observed using the original GCSI; however, different calculation in SA and in the geometric mean allows to capture the V-shaped cumulative GC graph for *Methanococcus aeolicus *more correctly with the aforementioned score of 0.107, whereas it was 0.071 with the original GCSI. Moreover, the new index allows the inclusion of small genomes such as that of *Mycoplasma genitalium *to the analysis because of fixed window numbers, and the availability of z-score clearly elucidates the significant gGCSI.

Note that the gGCSI is a measure of the clarity of V-shape cumulative GC skew. A high gGCSI score suggests strong mutation or selection pressures induced by bi-directional replication machinery starting from a single origin, whereas a low gGCSI score does not necessarily imply the existence of alternative replication machinery such as multiple replication origins. Weak GC skew can also result from long doubling times, as exemplified by low gGCSI scores in *Mycoplasma *and *Cyanobacteria *species. It is also worth noting that the gGCSI and z-score are weakly correlated (r = 0.578 and *ρ *= 0.678). Since z-score is calculated from the distribution of gGCSI values calculated for randomly shuffled genome sequences for 100 iterations, this value indicates the non-randomness of the observed gGCSI. Therefore, the correlation between the gGCSI score and its z-score indicates that high degree of skewness is not a random property that can happen by chance or due to certain bias in the genome such as extremely high GC content, and that certain mutation or selective pressure was required to shape the pronounced GC skew. Prediction of replication origins can be erroneous in species where GC skew is not clear or where multiple origins exist. gGCSI can thus be used as a confidence measure for GC skew-based predictions; according to the above results, chromosomes with gGCSI > 0.1 and z-score > 3 can be considered to have sufficient GC skew strength for accurate prediction with this number of windows.

### Difference in GC skew strength between plasmids with different types of replication machinery

**Figure 3:**
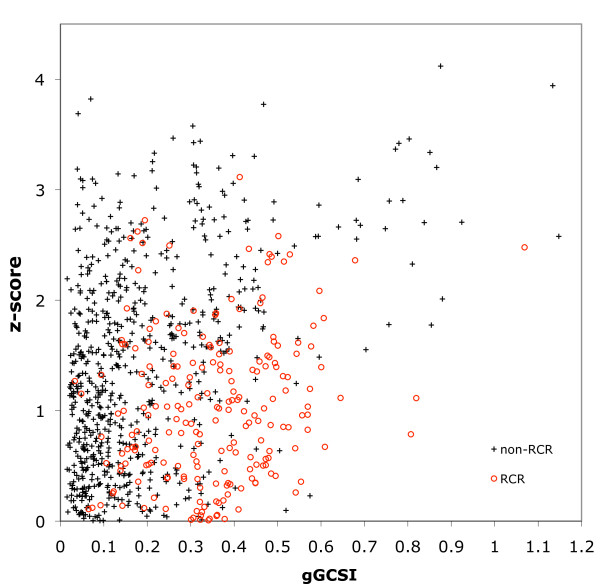
**Difference in GC skew strength between RCR and non-RCR plasmids**. Plot of gGCSI (X-axis) and z-score (Y-axis) for 211 RCR (red circles) and 697 non-RCR (black crosses) plasmids with 64 windows. Most RCR plasmids have gGCSI > 0.1 and z-score < 2, whereas non-RCR replicons show correlation in z-score and gGCSI.

We tested the distribution of gGCSI values in 908 bacterial plasmids using 64 windows to match the smaller size of these genomes (Figure 3). Of the 908 plasmids, 697 were putative non-RCR replicons as determined by their lack of the RCR initiator Rep protein [25], and 211 were RCR plasmids obtained from the Database of Plasmid Replicons [42]. The 697 non-RCR plasmids showed a similar score distribution to those of bacterial genomes shown in Figure 2, with a correlation between the gGCSI and z-score (r = 0.420 and *ρ *= 0.355). The RCR plasmids were distributed differently from the non-RCR plasmids (median of 0.134), mostly having high gGCSI values (median of 0.357) that were correlated with their z-scores (r = 0.195 and *ρ *= 0.182). As stated earlier, because RCR is based on strand displacement, one strand of the duplex DNA always serves as the template for replication, presumably resulting in continuous G/C bias along the entire genome without any shift point. This leads to high *dist *values concurrent with low *SA *values. Whereas the resulting geometric mean of these values (i.e., the gGCSI) becomes relatively high because one of the two values is high, the z-score remains low, because randomising the sequence will yield similar levels of *SA *and *dist *values and, subsequently, a similar gGCSI. This characteristic distribution is observable in Figure 3, where the RCR plasmids are mostly distributed below the non-RCR plasmids, with insignificant z-scores (p > 0.01 for z < 2.33) and relatively high but narrowly distributed gGCSI scores.

### Correlation of GC skew strength between plasmids and their hosts

**Figure 4:**
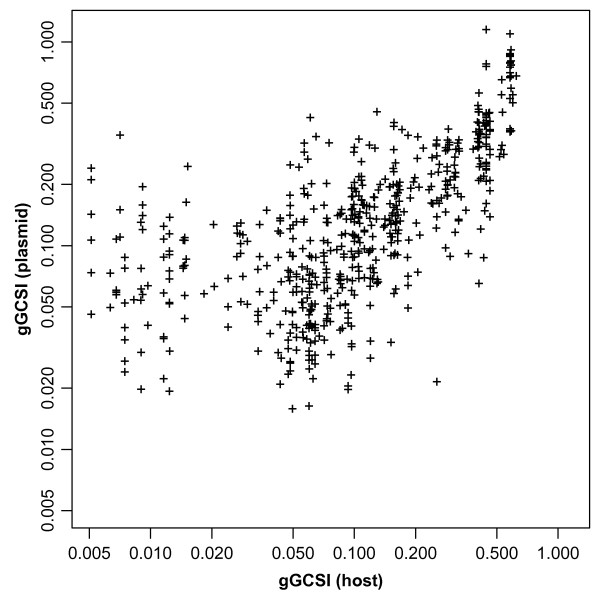
**Correlation of GC skew strength between plasmids and hosts**. Log-log plot of gGCSI of plasmids against that of their corresponding host chromosomes.

In order to observe the effect of replication-related mutation or selection pressures on different replicons within the same cell, we analysed the correlation of gGCSI values between plasmids and chromosomes from the same bacterial strains. Plasmids are transferable replicons that are capable of autonomous replication. Although the size, nucleotide composition, and available copy number of plasmids depend on growth conditions and hosts, plasmids maintain a finite copy number per cell under specific growth conditions in a specific host. Copy number control of plasmids is regulated through self-encoded negative regulation mechanisms using antisense RNA or through repeated genomic sequence elements called iterons in order to retain sufficient partitioning upon host cell division and also to avoid overshooting so that the plasmid can stably co-exist within the host cell without metabolic overload [43]. Therefore, plasmid replication is generally in harmony with host cell growth and thus with replication of the host chromosome, suggesting the existence of similar selection pressure in this pair of genomic elements. Using 302 host chromosomes and the 606 plasmids harboured by these strains, we have plotted the plasmid-host pairs according to their respective gGCSI values calculated with 64 windows (Figure 4). Because many plasmids and host chromosomes showed low gGCSI scores < 0.2, a log-log plot clarified this correlation (r = 0.791 and *ρ *= 0.706). We also verified the consistency of results when using different numbers of windows (data not shown). Our results indicate that plasmids tend to have GC skew strength similar to that of their known host chromosomes.

Previous work has shown similarity in dinucleotide composition between plasmids and host chromosomes [30,31]. This similarity is assumed to be caused by host-specific mutation biases of replication machineries, but the exact mechanisms remain unknown. Our finding that plasmids tend to be similar in GC skew strength to their host chromosomes strongly supports the assumption that host-specific properties of replication machineries homogenise the nucleotide composition of replicons in the cell.

### Application of gGCSI to other genomic compositional skews

This manuscript has thus far only considered the GC skew; however, other genomic compositional skews can be alternatively calculated using A+T, keto (G+T), or purine (A+G) bases, as AT skew (T-A)/(T+A), Keto skew (A+C-G-T)/(A+T+G+C), and Purine skew (C+T-A-G)/(A+T+G+C), respectively [44]. By utilizing these skew values as input instead of GC skew, we can likewise obtain gATSI, gKetoSI, and gPurineSI. In order to assess the applicability of these indices in comparison to the gGCSI, we have reproduced the Figures 2 to 4 using these indices (Supplemental Figures S4a-c, S5a-c, and S6a-c [see Additional File 1]). In all analyses, skew index with non-GC skews distributed in much narrower range, and separation of different replication machineries was best demonstrated with gGCSI. Correlation between the skew indices of the plasmids and their host chromosomes was also highest with gGCSI, with gGCSI (r = 0.791), ATSI (r = 0.491), gKetoSI (r = 0.569), and gPurineSI (r = 0.528).

### Implementation and availability

The algorithm described in this work is implemented as *gcsi *function in the 1.8.6 or above versions of G-language Genome Analysis Environment (G-language GAE) package [45-47], which includes the ability to calculate gATSI, gKetoSI, and gPurineSI along with gGCSI. G-language GAE is freely available with open source code licensed under GNU General Public License, Therefore, researchers can readily utilize gGCSI in their analyses through the Perl Application Programming Interface, or through web services provided by the G-language Project [48].

## Conclusions

Generalised GC skew index (gGCSI) is a quantitative measure of GC skew strength in genomes of any length that enables comparative study of replication-related mutation or selection pressures in bacterial chromosomes and plasmids. The gGCSI can be used to suggest the type of replication machinery used, i.e., bi-directional replication from a single origin and replication from multiple origins in eubacteria and archaea, as well as RCR in plasmids. The correlation of the degree of GC skew between bacterial plasmids and their host chromosomes suggests that these replicons within the same cells have replicated using the same replication machinery. gGCSI can be a useful measure for the study of replication-related features in bacterial genomes, and the index also provides confidence measures for GC skew-based predictions of replication origins.

## Methods

### Software and genome sequences

Genome analyses were conducted using the G-language Genome Analysis Environment version 1.8.6 [45-47], and gGCSI is implemented and released with this software package. The 846 complete chromosome sequences of eubacteria (710 strains, note that several strains contain multiple chromosomes) and archaea (53 strains) and 713 plasmid genomes were obtained from the NCBI FTP repository [49]. The 713 plasmids were further filtered to remove RCR replicons by excluding plasmids containing the RCR initiator protein Rep (COG5655: plasmid rolling circle replication initiator protein and truncated derivatives), leaving 697 genomes. A similarity search using BLASTP [50] with the 34 Rep sequences included in these genomes resulted in same number of filtered genomes. The 211 RCR plasmid genomes were downloaded through the links provided in the Database of Plasmid Replicons (DPR) [42]. For comparison of the strength in replication-related mutation or selection pressures between host chromosomes and plasmids, 302 chromosomes of host bacteria that harbour 606 plasmids were used.

### Calculation of the GCSI

The GCSI was calculated as the weighted arithmetic mean of *SR *and *dist*, as follows:

where *k*_1 _= 1/6000 and *k*_2 _= 1/600 were obtained from regression analysis of all available complete bacterial chromosomes. *SR *is the signal to noise ratio (S/N) of the 1-Hz power spectrum obtained from the fast Fourier transform (FFT) of the GC skew graph. FFT transforms a given signal to reveal the frequency components making up the input signal, which is computationally optimised by using powers of two for the window numbers. GC skew can be thought of as a discrete signal along the continuous axis of genomic position and FFT *F*(*k*) of a signal of length *N*, *f*(*n*), where *n *= 0, 1, ..., *N *-1, at frequency *k*, is calculated as follows:

where . The power spectrum *PS*(*k*) of *F*(*k*) was further defined as

at each frequency *k*. In this power spectrum, GC skew shows the greatest contributing component at 1-Hz frequency, corresponding to the two replichores having opposite polarity (discrete sine wave) [21]. S/N of the 1 Hz frequency, i.e., *SR*, is calculated as follows:

*dist *is calculated as the absolute difference between the maximum and minimum values of cumulative GC skew graph.

### Calculation of the gGCSI

The gGCSI is calculated as the weighted geometric mean of *SA *and *dist*(*norm*), as follows:

where *k*_1 _= 1/6000 and *k*_2 _= 1/600 as in GCSI. *SA *is the normalised spectral amplitude at 1-Hz, which is equivalent to *PS*(1).

where *k*_3 _= 600000, *k*_4 _= 40, and *α *= 0.4, as calculated by regression analysis.

Normalised *dist, dist*(*norm*), is calculated as follows:

where *W *is the number of windows used in the analysis.

### Calculation of *z*-score and *p*-value

Because the gGCSI is independent of the window size and number of windows, the significance of the gGCSI value should be noted to determine whether the number of windows used in the analysis is statistically sufficient to give the resulting value. Therefore, the significance measure is calculated from the distribution of gGCSI values for a shuffled input signal. For a given discrete GC skew signal *f*(*n*), 100 randomly shuffled series *f'*(*n*) are generated for which the gGCSI is calculated. Iteration size of 100 is chosen by default for computational efficiency, and this number can be configured when necessary. Then, the significance of the gGCSI based on the original GC skew signal *f*(*n*) is statistically assessed using the *z*-score based on the shuffled iterations, from which the *p*-value is obtained. Normal distribution of shuffled iterations was confirmed with Kolmovorov-Smirnov-Lillifors test with *p *< 0.001, for all genomes used in this work. Because re-sampling methods change the necessary window numbers/sizes and the coordination of genomic loci and because purely random values ignore the effects of diverse GC content, we have chosen this parametric statistic.

## List of abbreviations

*dist*: Euclidean distance; FFT: fast Fourier transform; GCSI: GC skew index; gATSI: generalised AT skew index; gGCSI: generalised GC skew index; gKetoSI: generalised; Keto skew index; gPurineSI: generalised Purine skew index; RCR: rolling circle replication; *SA*: spectral amplitude; *SR*: spectral ratio; S/N: signal to noise ratio; r: Pearson product moment correlation coefficient; *ρ*: Spearman rho rank correlation coefficient.

## Authors' contributions

KA developed and validated the gGCSI and carried out the analysis with RCR plasmids. HS analysed the correlation of gGCSI of plasmids and their host chromosomes. MT supervised the project. All authors have read and approved the final manuscript.

## Supplementary Material

Additional file 1 Supplemental Figures Click here for file
