# Supplementary material for: Quantitative analysis of replication-related mutation and selection pressures in bacterial chromosomes and plasmids using generalised GC skew index
Source: BMC Genomics. 2009 Dec 30;10:640. doi: 10.1186/1471-2164-10-640 (PMC2804667; doi:10.1186/1471-2164-10-640)
Supplement: Additional file 1 — Supplemental Figures [file 1471-2164-10-640-S1.PDF]

Supplemental Figures

Figure S1 - Distribution of the optimal number of windows, window sizes, and genome sizes for the identification of optimal window size for gGCSI.

Figure S2 - Correlation of *SA* and *dist*.

Figure S3 - Cumulative GC skew graph of *Methanococcus aeolicus*

Figure S4 - Difference in base composition skew strength between eubacteria and archaea using gATSI, gKetoSI, and gPurineSI.

Figure S5 - Difference in base composition skew strength between RCR and non-RCR plasmids using gATSI, gKetoSI, and gPurineSI.

Figure S6 - Correlation of base composition skew strength between plasmids and hosts using gATSI, gKetoSI, and gPurineSI.

optimalwindow: Distribution

Distributions

number of windows | window size (bp) | genome size (bp)

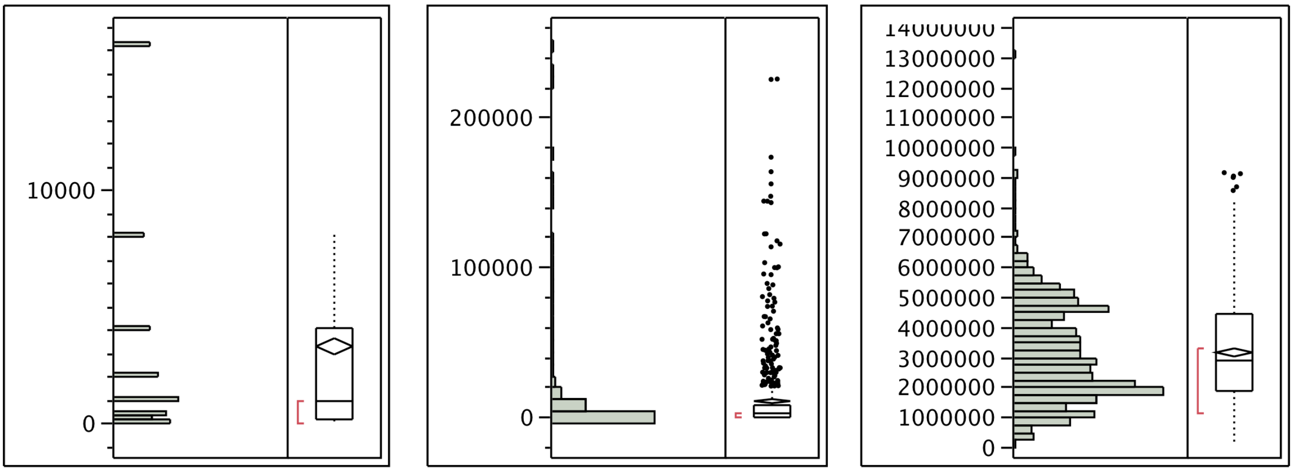

number of windows

Quantiles

100.0%	maximum	16384
99.5%		16384
97.5%		16384
90.0%		16384
75.0%	quartile	4096
50.0%	median	1024
25.0%	quartile	256
10.0%		128
2.5%		32
0.5%		16
0.0%	minimum	16

Moments

Mean	3327.4313
Std Dev	4937.4848
Std Err Mean	169.95523
upper 95% Mean	3661.0164
lower 95% Mean	2993.8462
N	844

window size (bp)

Quantiles

100.0%	maximum	249838
99.5%		213304
97.5%		95159
90.0%		26113
75.0%	quartile	8618
50.0%	median	2511
25.0%	quartile	734
10.0%		280
2.5%		128
0.5%		80
0.0%	minimum	52

Moments

Mean	11174.716
Std Dev	26832.606
Std Err Mean	923.61639
upper 95% Mean	12987.573
lower 95% Mean	9361.858
N	844

genome size (bp)

Quantiles

100.0%	maximum	13033779
99.5%		9087778.5
97.5%		6751178.1
90.0%		5312027
75.0%	quartile	4492712.3
50.0%	median	2886167.5
25.0%	quartile	1864797.8
10.0%		1196890.5
2.5%		698703.13
0.5%		280257.38
0.0%	minimum	159662

Moments

Mean	3178843.3
Std Dev	1706052
Std Err Mean	58724.731
upper 95% Mean	3294107.2
lower 95% Mean	3063579.5
N	844

Figure S1 - Distribution of the optimal number of windows, window sizes, and genome sizes for the identification of optimal window size for gGCSI.

In order to identify the optimal window size, we calculated gGCSI using number of windows from 8 to 32768 in all bacterial genomes used in this work, and identified the windows size where the change in gGCSI value is minimum compared to adjacent window counts. For example, in Table 1 (example with *E.coli*), window number of 4096 has the least difference with the next window counts (0.0001 difference with 2048 windows and 0.0003 difference with 8192 windows). As shown in this figure, the median of optimal window number in all bacteria is 1024, which corresponds to the median of 2511bp/window. Therefore, if a genome is sufficiently large, use of 1024 windows (2511bp/window) produces the most accurate gGCSI value.

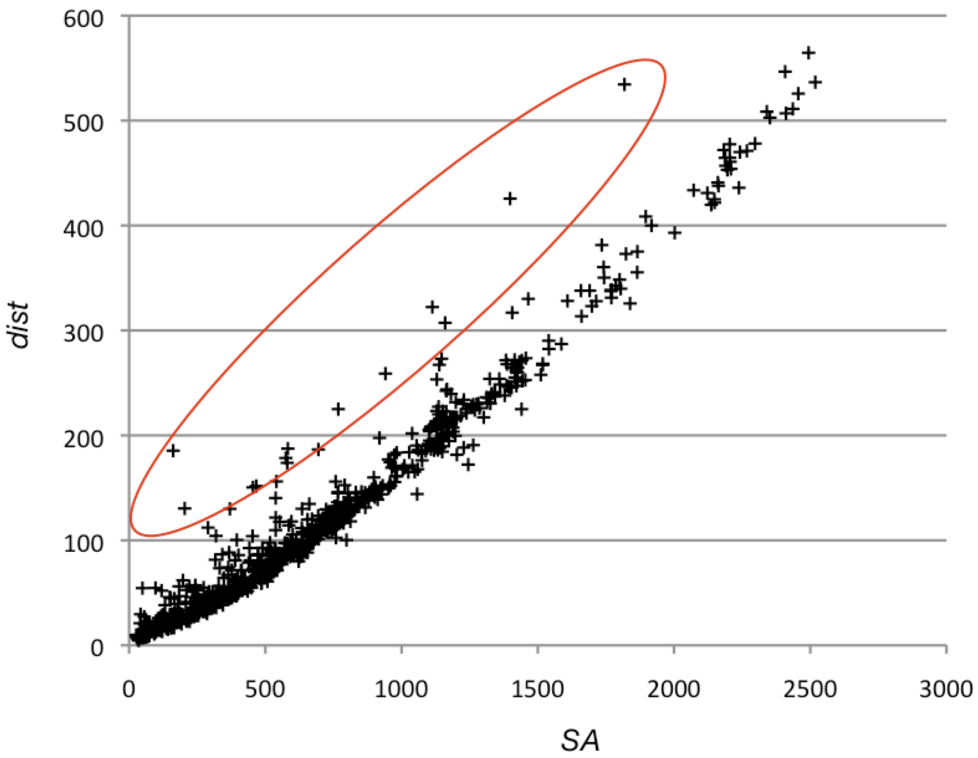

Figure S2 - Correlation of *SA* and *dist*.

SA and *dist* are generally correlated, and majority of the genomes exhibit *dist*/*SA* ratio of around 0.184. However, this ratio varies by about 10-fold among the genomes, so that the geometric mean better captures the balance between the two indices than the arithmetic mean: (10x + x)/2 = 5.5x, whereas sqrt(10x * x) ≈ 3.3x. When GC skew continuously exists along one strand of the genome and does not shift its polarity, the strand results in extremely high *dist* while *SA* is low, deviating from the above *dist*/*SA* ratio. The genomes of *Pseudoalteromonas haloplanktis* TAC125 and *Halorhodospira halophila* SL1 (included in the region marked by red oval) are good examples for such continuously biased genomes, that show gGCSI < 0.1 with geometric mean, but exceed this threshold when calculated with arithmetic mean. This deviation is more pronounced with RCR plasmids that have the same non-shifting GC skew. 16 RCR plasmids used in this work showed gGCSI > 1.0 (with maximum of 1.544) when calculated with arithmetic mean, but the use of geometric mean limits to only one genome exceeding gGCSI > 1.0, with 1.069.

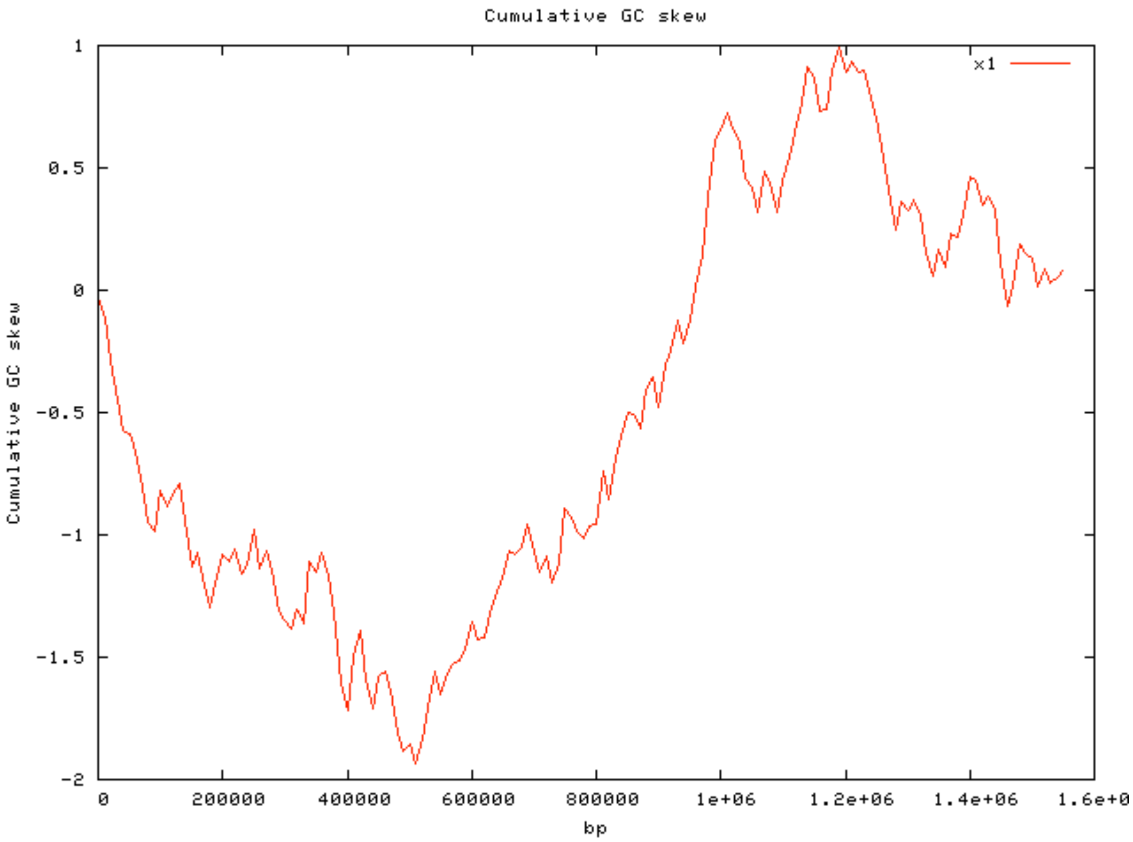

Figure S3 - Cumulative GC skew graph of *Methanococcus aeolicus*

Although *Methanococcus aeolicus* (gGCSI = 0.107, z = 4.62) has no published evidence suggesting or confirming a single origin of replication, its gGCSI score suggests a high likelihood of bi-directional replication, which is supported by this V-shaped cumulative GC skew graph

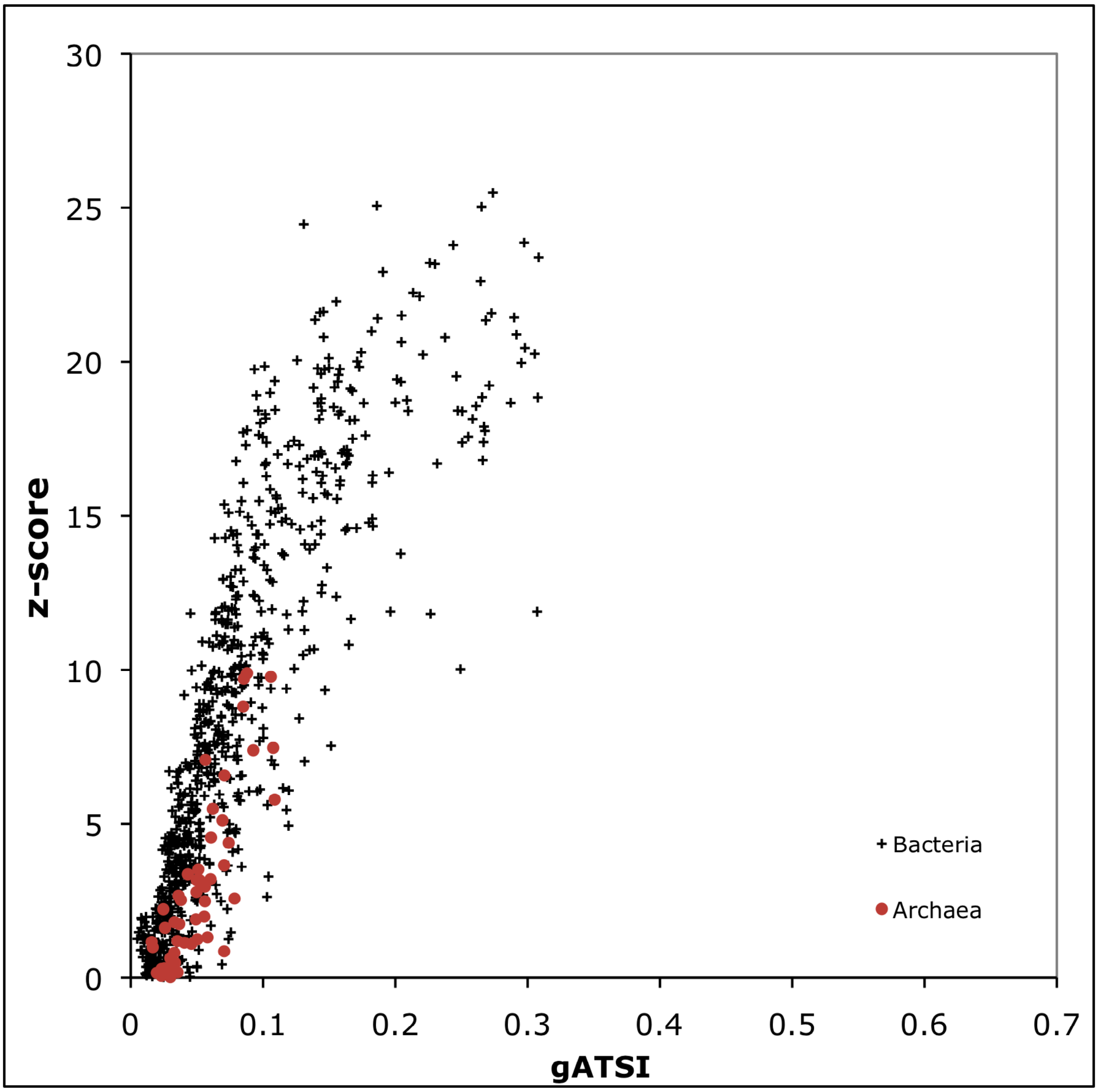

Figure S4a - Difference in base composition skew strength between eubacteria and archaea using gATSI.

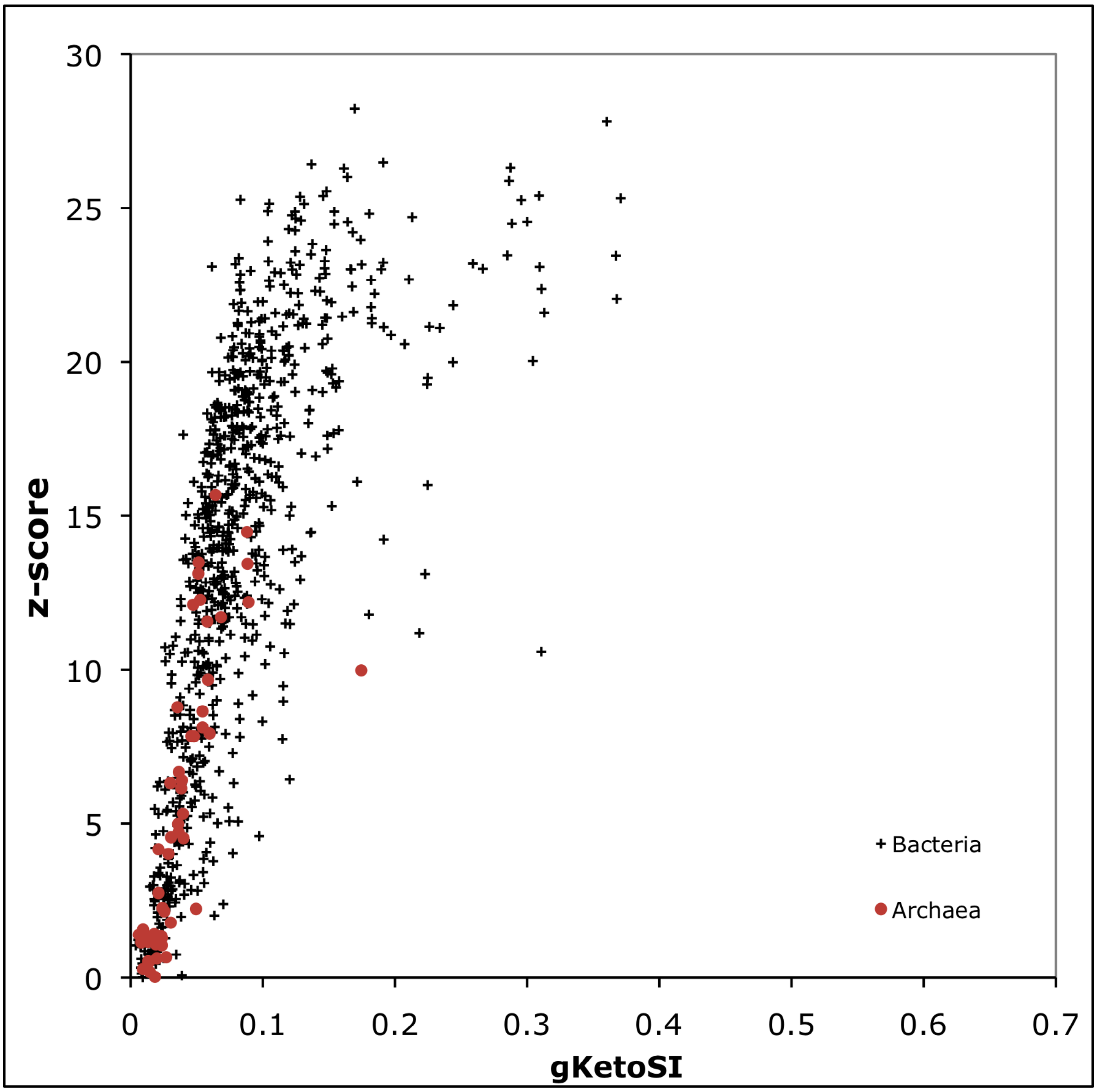

Figure S4b - Difference in base composition skew strength between eubacteria and archaea using gKetoSI.

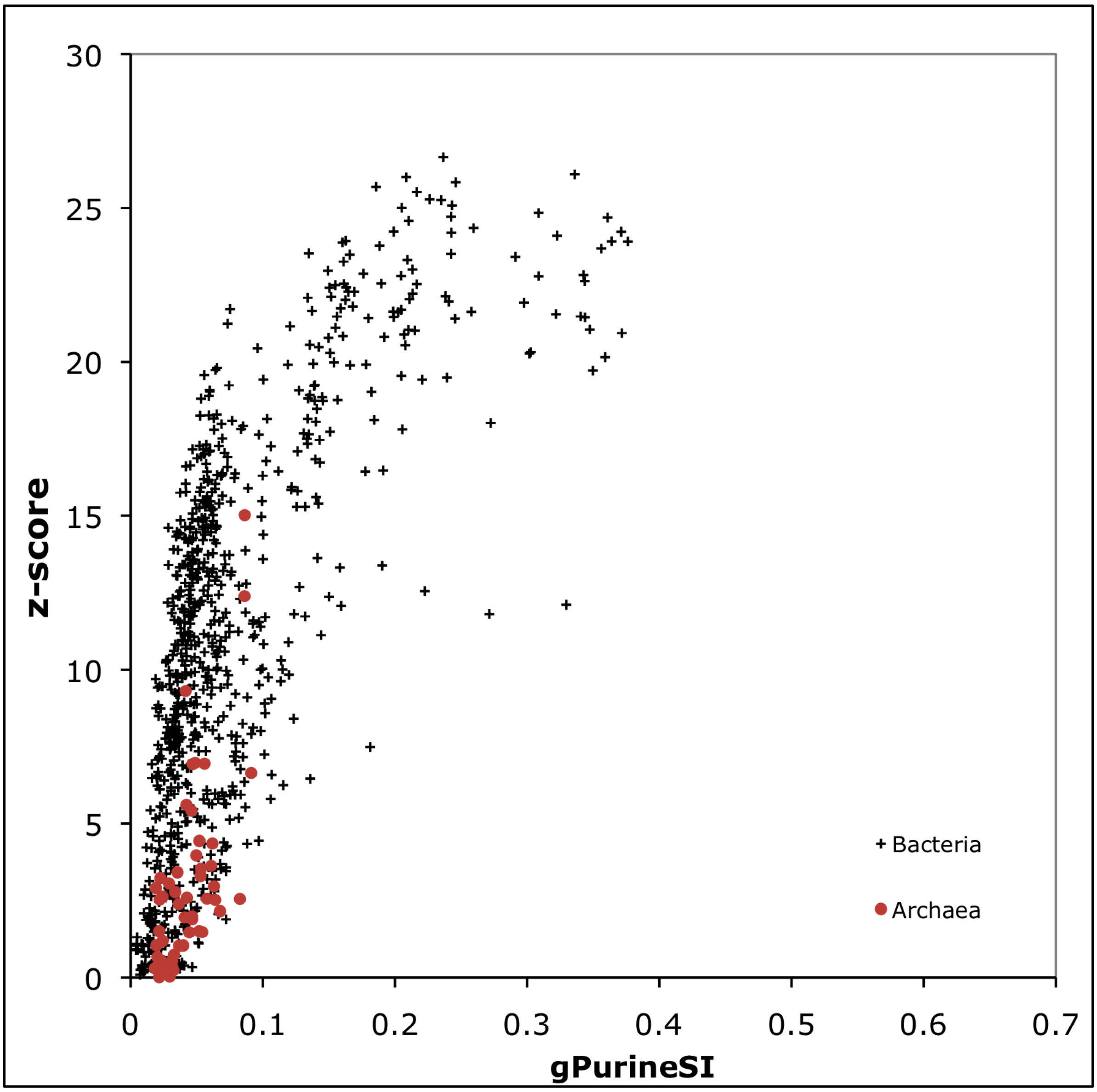

Figure S4c - Difference in base composition skew strength between eubacteria and archaea using gPurineSI.

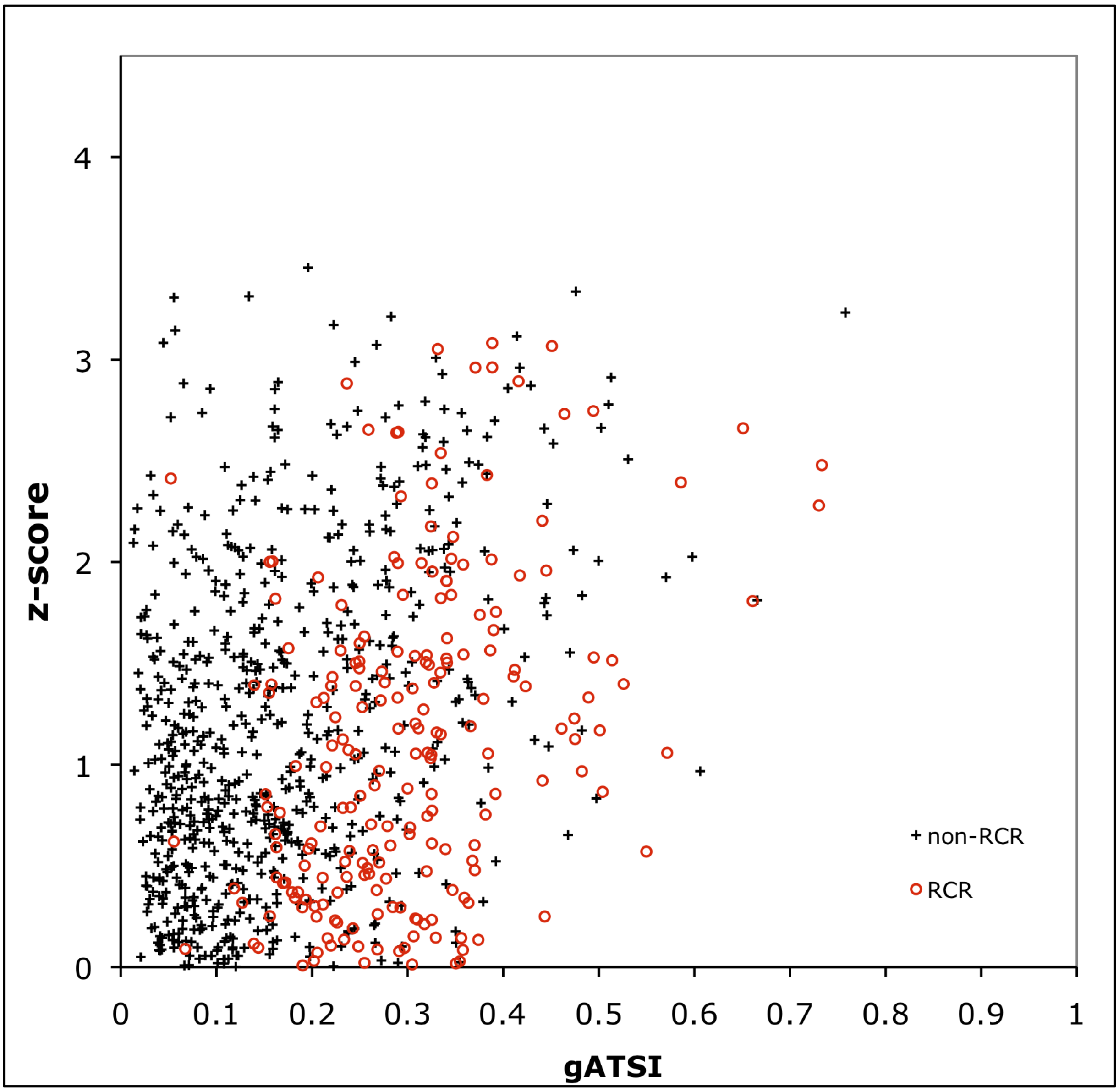

Figure S5a - Difference in base composition skew strength between RCR and non-RCR plasmids using gATSI.

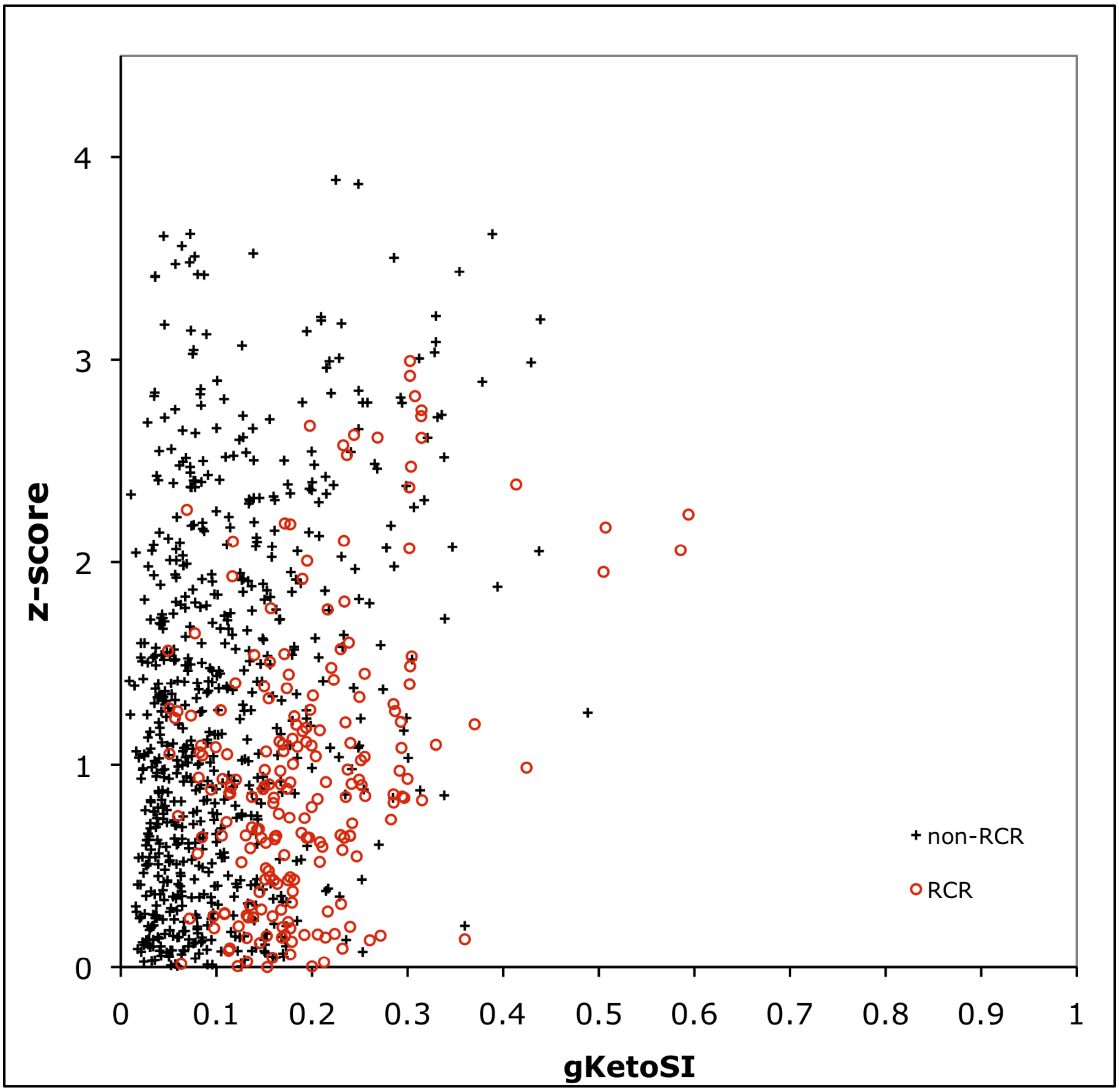

Figure S5b - Difference in base composition skew strength between RCR and non-RCR plasmids using gKetoSI.

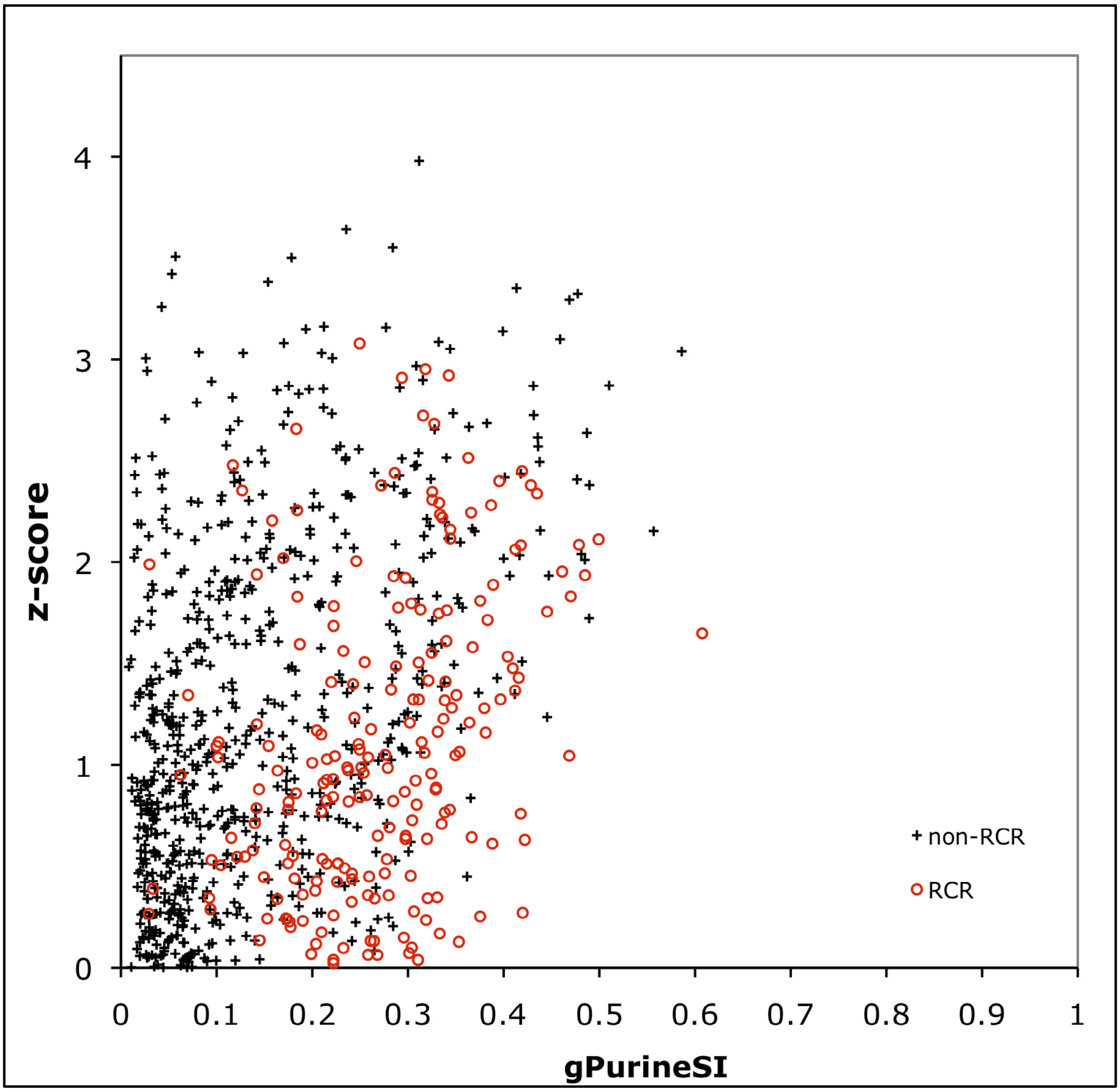

Figure S5c - Difference in base composition skew strength between RCR and non-RCR plasmids using gPurineSI.

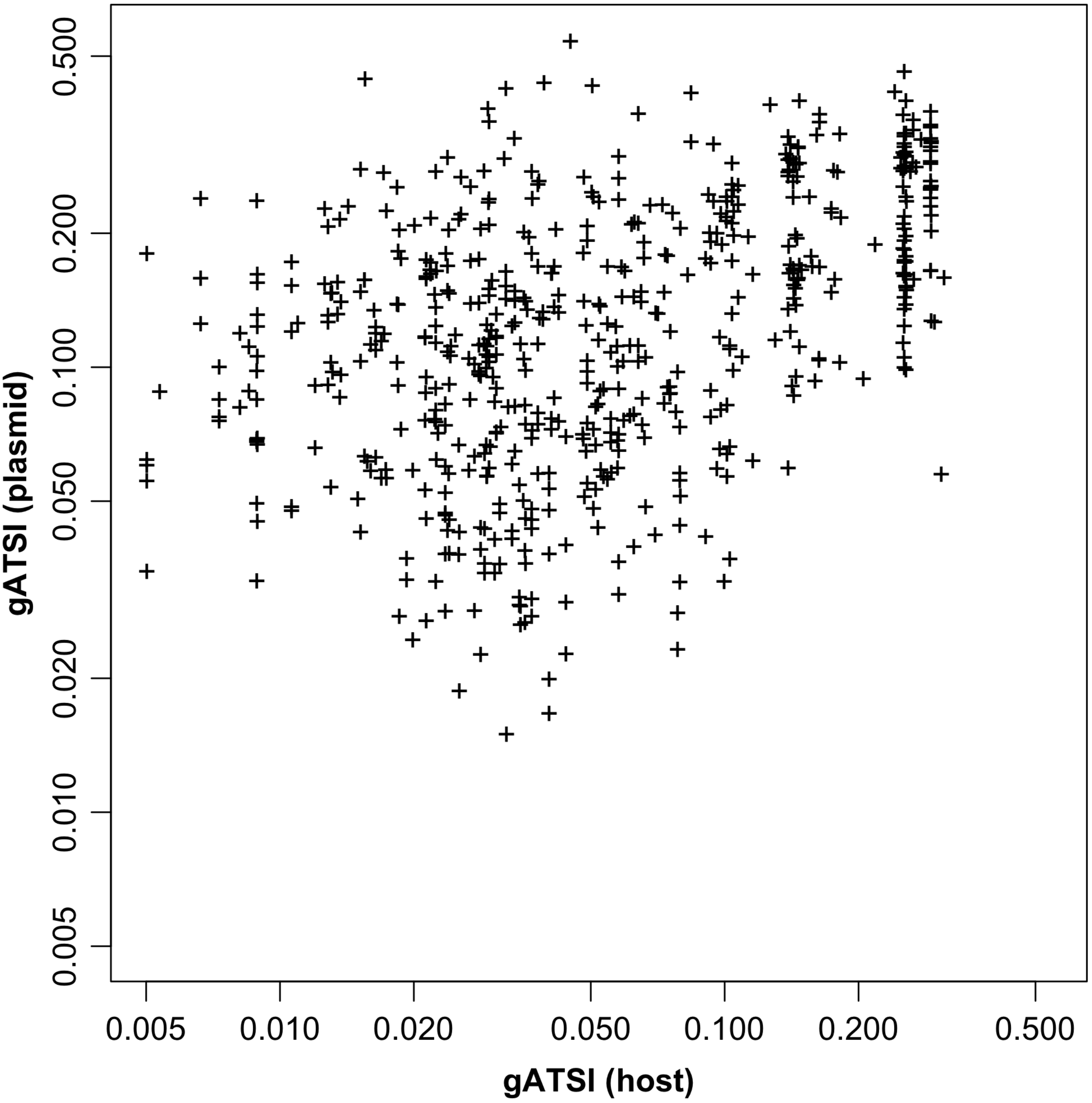

Figure S6a - Correlation of base composition skew strength between plasmids and hosts using gATSI.

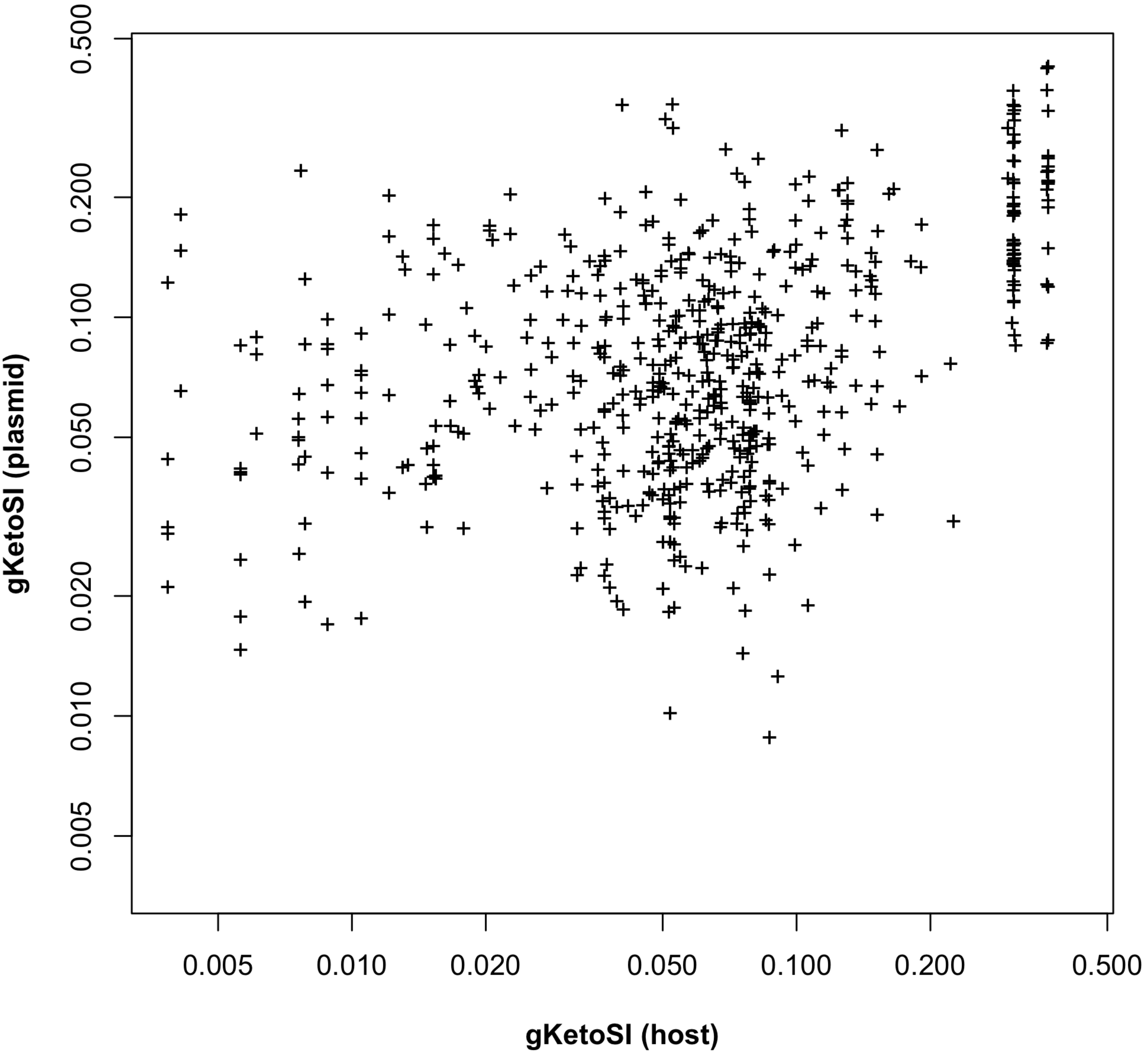

Figure S6b - Correlation of base composition skew strength between plasmids and hosts using gKetoSI.

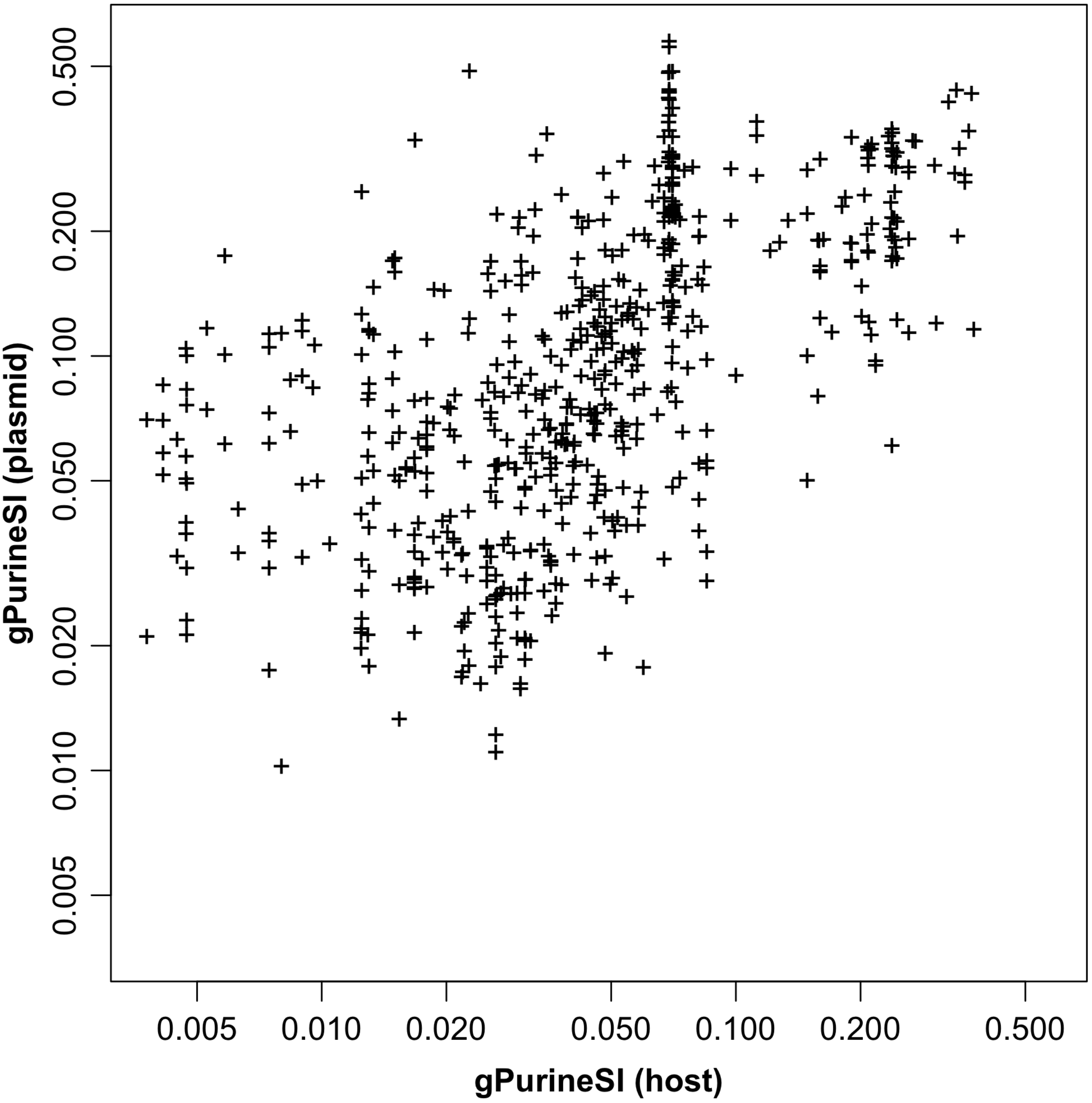

Figure S6c - Correlation of base composition skew strength between plasmids and hosts using gPurineSI.
